# Uncovering the Drivers of Childhood Immunization Inequality with Caregivers, Community Members and Health System Stakeholders: Results from a Human-Centered Design Study in DRC, Mozambique and Nigeria

**DOI:** 10.3390/vaccines11030689

**Published:** 2023-03-17

**Authors:** Jessica C. Shearer, Olivia Nava, Wendy Prosser, Saira Nawaz, Salva Mulongo, Thérèse Mambu, Eric Mafuta, Khatia Munguambe, Betuel Sigauque, Yakubu Joel Cherima, Olawale Durosinmi-Etti, Obehi Okojie, Idris Suleman Hadejia, Femi Oyewole, Dessie Ayalew Mekonnen, Natasha Kanagat, Carol Hooks, Rebecca Fields, Vanessa Richart, Grace Chee

**Affiliations:** 1 PATH USA, Seattle, WA 98102, USA; 2 Independent Consultant, Oakland, CA 94608, USA; 3 JSI Research and Training Institute USA, Arlington, VA 22202, USA; 4 PATH DRC, Kinshasa 7525, Democratic Republic of the Congo; 5 Kinshasa School of Public Health, University of Kinshasa, Kinshasa 11, Democratic Republic of the Congo; 6 Community Health Department, Eduardo Mondlane University, Maputo 1102, Mozambique; 7 JSI Research and Training Institute Mozambique, Maputo, Mozambique; 8 JSI Research and Training Institute Nigeria, Abuja, Nigeria; 9 Department of Community Health, University of Benin, Benin City 300271, Nigeria; 10 Department of Community Medicine, Ahmadu Bello University, Zaria 810107, Nigeria; 11 Consultant Public Health Physician, Lagos, Nigeria; 12 Manoff Group, Washington, DC 20008, USA

**Keywords:** health inequalities, immunization, equity, inequality, human-centered design, vaccination services, zero-dose children, community

## Abstract

Background: The importance of immunization for child survival underscores the need to eliminate immunization inequalities. Few existing studies of inequalities use approaches that view the challenges and potential solutions from the perspective of caregivers. This study aimed to identify barriers and context-appropriate solutions by engaging deeply with caregivers, community members, health workers, and other health system actors through participatory action research, intersectionality, and human-centered design lenses. Methods: This study was conducted in the Demographic Republic of Congo, Mozambique and Nigeria. Rapid qualitative research was followed by co-creation workshops with study participants to identify solutions. We analyzed the data using the UNICEF Journey to Health and Immunization Framework. Results: Caregivers of zero-dose and under-immunized children faced multiple intersecting and interacting barriers related to gender, poverty, geographic access, and service experience. Immunization programs were not aligned with needs of the most vulnerable due to the sub-optimal implementation of pro-equity strategies, such as outreach vaccination. Caregivers and communities identified feasible solutions through co-creation workshops and this approach should be used whenever possible to inform local planning. Conclusions: Policymakers and managers can integrate HCD and intersectionality mindsets into existing planning and assessment processes, and focus on overcoming root causes of sub-optimal implementation.

## 1. Introduction

Immunization is widely recognized as one of the most important public health interventions for reducing childhood morbidity and mortality [1]. Enormous efforts led to a significant increase in global coverage of the third dose of diphtheria–tetanus–pertussis (DTP3) vaccine over the past two decades, reaching its highest point at 86% in 2019, although this fell to 83% in 2020 due to the COVID-19 pandemic, and 81% in 2021 [2]. Immunization services miss millions of children each year, including those who are not fully vaccinated and those considered zero-dose, defined as not having received the first dose of a DTP-containing vaccine. It is estimated that 16 million children were zero-dose in 2020 and 18 million were zero-dose in 2021. These children are at risk of illness or death, and are likely to live in circumstances that further exacerbate this risk [2].

Research exists on the drivers of immunization inequality [3,4,5,6,7,8,9,10], yet most of the existing research focuses either on individual attributes or health system drivers, without analysis of the social and structural processes that produce inequalities [11]. Recent attention to the role of gender in immunization (in)equity is overdue [8,12] but too often, gender is explored alone, without consideration of how it intersects and interacts with other social, institutional, and structural dimensions of inequality, including social determinants of health. Novel research approaches are needed to reconceptualize immunization inequality—and potential solutions to overcoming it—from caregivers’ lived perspectives.

This study seeks a way forward to engage and support individuals, caregivers, families, communities, and the health system to co-produce immunization equity. We apply paradigms and approaches from a human-centered design (HCD) and intersectionality [11,13], to reconceptualize the barriers, facilitators, and root causes of no immunization and under-immunization from the perspective of caregivers of infants and young children. When applied to health, intersectionality is the theory that individuals’ lives are shaped by multiple social, institutional, and structural axes, that work together and interact to produce advantages or disadvantages [11,13]. Through this lens, we shift towards understanding each caregivers’ experience as unique to their situation and the role that all social forces play in empowering or disempowering them. HCD has been used in global health programs to better understand the needs and context of end-users, and to co-create context-appropriate solutions [14], which this study sought to do. In all steps of the study’s design and implementation, we sought to increase empathy for caregivers, an important step towards equity and justice.

### 1.1. Country Contexts

#### 1.1.1. Democratic Republic of the Congo

The DRC remains among the most vulnerable countries in the world for the spread of vaccine-preventable diseases, as evidenced by the most recent outbreaks in measles, polio and yellow fever. DRC is home to poor infrastructure and difficult access, as well as remote missed communities, mobile and conflict-affected populations, and weak health systems, all leading to a high proportion of zero-dose and under-immunized children, and substantial within-country inequalities. In 2021, the WHO and UNICEF estimated that more than 700,000 children in the DRC were zero-dose [2]. Causes of no immunization and under-immunization include frequent vaccine stockouts, weak funding and governance, limited and demotivated human resources, poor service experience [15], and access difficulties. Analyses of the 2013 Demographic and Health Survey (DHS) survey data indicate a DTP3 coverage gap of more than 20-percent points between the lowest and highest wealth quintiles, and between rural and urban residents [6].

#### 1.1.2. Mozambique

Mozambique has a history of political commitment to vaccination and primary health care; however the WUENIC estimate of DTP3 coverage dropped from 88% in 2019, to 79% in 2020, to 61% in 2021, in large part due to vaccine stockouts. Equity analyses of the 2015 DHS data show that while the wealth gap has narrowed since 1997, there remains a 20-percent point difference between the lowest and highest wealth quintiles. Children of educated mothers and mothers living in urban settings have DTP3 coverage approximately 15 percentage points higher than uneducated mothers or those living in rural areas [6]. Mozambique also has substantial within-country geographic inequalities, with a DTP3 ranging from 74.6% in Nampula, to 97.5% in Maputo Province in the 2015 DHS survey [16]. Evidence suggests that the general public and caregivers of young children, in particular, have positive attitudes toward child vaccination [17]. However, long travel distances, frequent stockouts, the perception of adverse outcomes from administering multiple vaccines at the same time and fear of being mistreated by healthcare providers contribute to low vaccine coverage and wide geographic inequalities [17,18,19].

#### 1.1.3. Nigeria

In 2021, Nigeria had one of the highest numbers of zero-dose children (2.2 million) in the world [2], a challenge that was exacerbated by COVID-19. Equity analyses of 2013 and 2018 DHS data demonstrate stark inequalities based on wealth (over 70 percentage points), maternal education (50 percentage points), and place of residence (nearly 40 percentage points). Nigeria is one of the few countries where, nationally, more boys are vaccinated than girls [6]. Geographic inequality is significant between Nigeria’s states, and within them, the majority of zero-dose and under-immunized children are living in the northern states. Qualitative research of barriers to vaccination has highlighted mistrust of the government and vaccines, lack of awareness, fear of adverse events following immunization, shortage of health workers, long waiting times, and long travel times [20]. COVID-19 has also exacerbated some of these barriers and recent research on the reasons behind the slow uptake of COVID-19 vaccinations point to many of the same determinants [21].

## 2. Materials and Methods

### 2.1. Study Design

Each country implemented a qualitative study to identify the barriers and facilitators of vaccination faced by caregivers of zero-dose and under-immunized children, and to identify context-tailored solutions from the perspective of caregivers and other stakeholders. This study was implemented as part of the USAID-funded MOMENTUM Routine Immunization Transformation and Equity project, and a major objective of this study was to inform the design of locally tailored solutions to include in the project’s activity workplans. We drew from participatory action research, intersectionality, and human-centered design (HCD) approaches to design a study that would ensure the engagement and collaboration of stakeholders at all levels. A key innovation was the inclusion of community-based co-creation workshops, which sought to validate initial study findings, build empathy for caregivers among other stakeholders, and use HCD tools to identify potential interventions to overcome barriers. Our approach aligns with draft guidance from UNICEF on integrating HCD into sub-national immunization coverage and equity assessments [22].

#### 2.1.1. Analytical Framework

**Figure 1:**
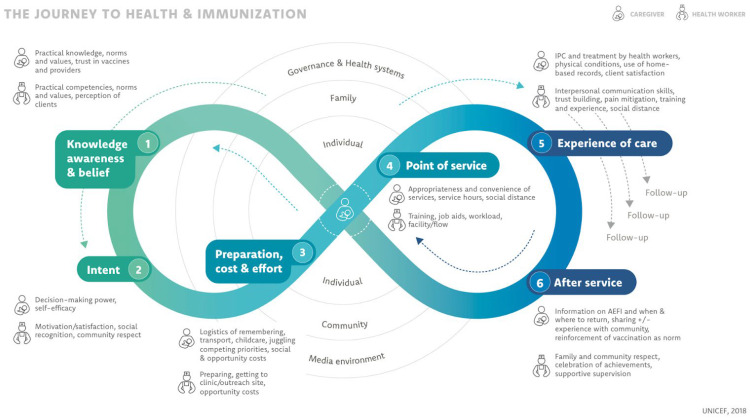
The UNICEF Journey to Health and Immunization framework. UNICEF, Demand for health services field guide: a human-centered approach. New York: UNICEF, 2018.

The research team drew on three similar analytical frameworks to inform research questions and data collection tools: the UNICEF Journey to Health and Immunization framework [22]; the WHO Behavioural and Social Drivers framework [23]; and the Determinants of childhood vaccine coverage model [5,24]. These frameworks all explain vaccine uptake as a function of three main factors: behavioral drivers such as knowledge, awareness and beliefs, attitudes, and social norms; the practical and access issues caregivers face, including geographic and financial access; and characteristics of the health system, such as service convenience, quality, and experience of care. We used the UNICEF framework to guide analysis as it most closely represents empathy for a caregiver and concepts of HCD and intersectionality, by situating them in the center of an ecosystem [25] and explicitly recognizing the influence of multiple levels (Figure 1). This framework also captures issues faced by health personnel.

These models and our pilot phase forced us to clarify the meaning of ‘caregiver’ in our study countries. In the DRC, Mozambique, and Nigeria, we observed that mothers, or occasionally other adult female family members, had the socially prescribed role to get a child vaccinated, and so we use the term ‘caregiver’ in this paper with an understanding that the data primarily reflects the experiences of mothers. We acknowledge that caregivers could be men, including fathers, but these findings specifically reflect the gendered realities of the female caregivers we interacted with.

#### 2.1.2. Study Setting and Sampling Criteria

The study was implemented in the DRC (May–June 2021), Mozambique (July–August 2021), and Nigeria (October 2022) by research teams comprising qualitative researchers and project staff with expertise in immunization. The selection of study states/provinces, districts, health facilities, and communities was done stepwise with health system managers at each level. As a qualitative study, we did not use sample size calculations, but did aim to interview enough respondents to achieve theoretical saturation on our research questions. The first sub-national unit, states or provinces, were selected with the project funder and national immunization managers, to ensure that this project’s eventual interventions were implemented in provinces in need of an immunization technical partner. Within those states/provinces, we used survey or administrative data to rank districts by their proportion of un- and under-vaccinated children. Among those districts with the highest proportion of un- and under-vaccinated children, we purposefully selected districts that were accessible to our study team during the study period (all countries), represented a mix of both urban and rural districts (DRC), and were free from security concerns (Nigeria). The number of districts selected in each country depended on available financial and human resources to implement the study, resulting in seven districts (health zones) in the DRC, six in Mozambique, and three in Nigeria. Within the selected districts, we worked with district-level managers to select health facilities that represented typical cases of that district, and then selected one community linked to each facility with high proportions of zero-dose or missed communities. We worked with community leaders and CHWs to identify mothers of zero-dose and under-vaccinated children in selected communities.

### 2.2. Data Collection and Analysis, including Co-Creation Workshops

#### 2.2.1. Data Collection

The research team and additionally trained data collectors collected data for this study through semi-structured and open-ended in-depth interviews (IDIs), focus group discussions (FGDs; Nigeria only), and co-creation workshops. In Nigeria, FGDs were used instead of interviews at the community level, with caregivers and community members separately, to optimize time and resources. Interview and FGD guides reflected the analytical frameworks, and we adapted during implementation based on emerging insights. The teams also collected health facility data for common indicators of facility readiness and performance, as found in existing health facility surveys and immunization-specific supportive supervision checklists. While these quantitative data were later used to inform our project’s intervention design, we did not include them in the analysis presented at co-creation workshops due to the rapid implementation timelines (1–2 weeks for data collection, analysis, and co-creation) of these qualitative studies; thus, we do not report them here.

#### 2.2.2. Data Analysis and Synthesis

The research teams took notes during interviews and FGDs and wrote memos summarizing the key findings of each interview. Teams met daily in-person or online to discuss new data, emerging themes, and questions to probe further on. Upon completion of data collection in a study area, the teams rapidly sorted the data, key insights, and emerging themes into text-based tables, categorized according to the domains of the analytical frameworks. This step helped to identify key barriers and facilitators to vaccination in that community. Through this process, the research team selected three key issues to explore further during the local co-creation workshops, with the goals of strengthening participants’ empathy for caregivers by highlighting the challenges they face, and identifying common barriers that had the potential to be resolved through local solutions. The research team developed ‘personas’—short stories centered around a caregivers’ experiences based on the synthesized data—to illustrate each selected challenge at the co-creation workshop, while ensuring the confidentiality of the research participants. Following all data collection and workshops, country research teams synthesized all analyzed data into reports which informed the development of the project’s activity workplan.

#### 2.2.3. Co-Creation Workshops

Immediately following data collection, the research teams facilitated workshops with study participants and other relevant stakeholders at the district level. These co-creation workshops aimed to validate emerging findings from the data collection phase, strengthen feelings of empathy for caregivers, and identify locally relevant interventions to overcome identified barriers. The research team first summarized findings and facilitated discussion, and then implemented HCD tools adapted by the project to achieve the empathy and solution-identification objectives: the mothers’ vaccination ecosystem; solution briefs; and a solutioning activity (Table 1). All study participants were invited and workshops were attended by 20–30 individuals, including caregivers. Experienced facilitators were attentive to the possibly negative consequences of mixing multiple levels of social power, and took care to ensure the respect for and confidentiality of the caregiver attendees. Caregivers’ confidentiality was protected by the use of persona tools to share fictionalized findings based on the synthesis of experiences across all interviewees (Table 2). Community-level co-creation workshops were followed by district-level and then state/provincial and/or national workshops, to share insights and solutions from the level below, validate findings at each level, and assess motivation and priority for community-developed solutions across the other levels. Research teams generated additional insights on stakeholder motivations, preferences, and needs by observing the group discussions and taking notes.

#### 2.2.4. Cross-Country Synthesis

The research team reviewed country finding reports and manually re-coded data and findings according to the UNICEF framework and through the lens of intersectionality and power dynamics at the individual, institutional, and structural levels [13]. The team discussed key findings to better understand barriers and facilitators faced by caregivers and how they differed by context. This led to the identification of three mid-level themes which the team considered to be of broad importance and actionable, and emphasized the empathy mindset for caregivers and communities.

Ethical approval was granted from the Kinshasa School of Public Health (DRC), the University Eduardo Mondlane (Mozambique), and the Edo and Jigawa State Health and Research Ethics Committee (Nigeria). Consent to participate was obtained by investigators trained in ethical procedures and prior to any observation or engagement. Team members read the consent form to participants in the local language and provided time to ask questions and clarify concerns. The team obtained written or verbal consent (in some situations) after answering the participants’ questions and before beginning the activity or observation. As described in the consent form provided to the participants, all participants could request to withdraw from participation at any time.

## 3. Results

Table 3 summarizes the number of study participants by country and level of the health system. The section below presents synthesized findings from across the three countries, according to three emergent mid-level themes.

### 3.1. Social and Structural Factors Intersect to Produce Inequitable Power Relationships and Limit Health System Actors’ Empathy for Caregivers

Across all the countries, most of the caregivers interviewed expressed the desire for their child to be vaccinated, and most were aware of the general benefits of vaccines. However, gender, social factors, and structural inequalities intersected and interacted to produce a variety of barriers for caregivers (Table 4). The type and magnitude of these barriers differed by a caregivers’ social status, wealth, place of residence, and economic role—which in turn varied by country and region—and often played out as power dynamics that produced inequitable access to and quality of immunization services. Most caregivers reported some difficulty juggling their gender-prescribed tasks related to childcare and domestic work with getting a child vaccinated. These difficulties were more common among caregivers who faced other financial or time-related resource barriers, whether because of poverty or because the child’s father worked or lived away from the home. Gender inequality was sometimes apparent in the caregivers’ lack of agency to make a decision about whether to vaccinate her child. While most caregivers said they were able to make a decision themselves about vaccination, we also heard cases where caregivers noted they were not the key decision-makers, and this pertained more often to caregivers of zero-dose children. Decision-making divergence worked in both directions: sometimes women followed their male partners’ preference to not get vaccinated, and sometimes they followed his preference to get vaccinated. When decision-making agency intersected with wealth and women relied on their male partners for financial support to access vaccination, it often resulted in the child not getting vaccinated. When husbands assisted with practical aspects, such as childcare or transport, which was reported by some respondents, caregivers were more likely to seek vaccination. Gender dynamics were also presented in conversations related to adverse events following immunization (AEFI). Many caregivers reported that they feared AEFI, such as fever or fussiness, as an uncomfortable infant disrupted the household dynamic, and this fear increased if their husband had complained.

Equity-limiting power dynamics also existed within the health system, where health workers wielded power from relative privilege over clients, and experienced disempowerment from managers and institutions that did not value them. This created a vicious cycle of negative power relations between health workers and clients. We observed that caregivers of low socioeconomic status experienced more disrespectful care from health workers (all countries) and were most likely to be blamed for not vaccinating their children (DRC). These particular caregivers expressed feelings of shame for being inappropriately dressed (DRC, Mozambique). In the DRC and Mozambique, caregivers of zero-dose children felt a sense of shame or exclusion that prevented them from accessing services, and caregivers who experienced blame or disrespect at the vaccination facility were the least likely to return. Caregivers in the DRC and Nigeria reported having to pay illicit fees for vaccine services or cards, and transport costs, which resulted in some caregivers being unable to afford services. In the DRC these illicit fees were the consequence of health workers being unpaid; in Nigeria they were explained as necessary to run an underfunded system. Many caregivers reported being unable to overcome at least one cost-related barrier, whether the service fees, costs of transportation to reach the facility, or opportunity costs of leaving paid or unpaid work. In Nigeria, financial and non-financial incentives were given by partners to caregivers, to cover the opportunity cost and transport costs. In the DRC many respondents reported that they had appreciated receiving nutritional and other non-cash incentives in the past, and had lost trust in the health system when those incentives were ended. Cost barriers were most challenging for caregivers of zero-dose children and intersected with gender and other access barriers to limit vaccination (see Table 2 for caregiver personas used during the co-creation workshops to illustrate these intersections).

Negative beliefs or misinformation about vaccination or vaccines were rarely the sole barrier to vaccination, although they did exist and interact with other barriers in a caregivers’ overall influencing environment, particularly for the caregivers of zero-dose children. Interviews highlighted the critical role of religious leaders in influencing decisions—either towards or away from vaccines—in all countries. Our data suggest a lesser influence of CHWs or community health volunteers, often because they themselves did not have sufficient information on immunization services to counteract misinformation or provide practical information. In all communities, they faced retention and motivation challenges due to limited financing for community health, weakening their potential as a trusted link to the health system.

### 3.2. Insufficient Accountability, Governance, and Financing Respectively Contribute to Sub-Optimal Person-Centredness

At an institutional level, our findings indicated that the health systems faced multiple design and implementation constraints to fully delivering pro-equity or people-centered immunization and PHC services. Strategies to improve equity by aligning service design and delivery to the needs and preferences of those at greatest risk of access barriers—such as vaccination in communities (e.g., outreach or mobile vaccination services), expanded clinic hours, and community mobilization activities—existed in policy and facilities’ operational plans and budgets, but were sub-optimally implemented. The lack of person-centeredness was most acute for caregivers and communities that were geographically inaccessible, socially excluded, or faced financial access barriers. For example, many caregivers decided not to seek vaccination services because of the long, difficult, or unsafe walks to health centers, as well as long wait-times once there, and this was exacerbated among low-income women and those who were socially isolated. Planned outreach vaccination sessions are meant to overcome these barriers, but a theme across all three countries was dissatisfaction with the low frequency of these services or not knowing when they would occur. When community-based or outreach services were implemented, they were implemented in communities close to the health facility, as health workers faced their own challenges—financial or logistical—in reaching remote communities.

Respondents in all countries described insufficient or poorly planned and managed immunization and PHC budgets, which led to insufficient operational funds to implement these strategies. Triangulation of data across multiple levels of the health system identified the root causes of weak governance and accountability, insufficient and fragmented financial resources, and weak leadership and management capability. Poor resource generation, allocation, and management, thus, most affected communities that already faced access barriers to immunization services. In Mozambique and Nigeria, national policies supporting integrated health services were sub-optimally implemented due to insufficient and fragmented finances stemming from weak governance. In all countries there was a recognition that certain remote or migrant communities were missed entirely with health and social services, and that no mechanism existed to identify these communities and link them to services.

Current or previous experience with vaccine stockouts weakened caregivers’ trust in vaccination services. Many zero-dose children in the DRC were unvaccinated due to an ongoing shortage of BCG vaccines; the likelihood of a caregiver returning again after a missed opportunity for vaccination depended on the intersection of other barriers. Sub-optimal health worker motivation and performance was an important barrier to vaccination in all countries, ranging from absenteeism that led to missed opportunities for vaccination, to disrespectful care, to poor clinical quality of care. Caregiver reports of service quality varied across interviewees, but we noted that mothers of under-vaccinated children were likely to report poor service experience as a reason for not returning for additional doses. This included perceptions that sub-optimal clinical quality resulted in common side effects, such as swelling or sores at the vaccination site. As noted above, health workers themselves experienced institutional inequality and disempowerment. Despite these conditions, and as noted elsewhere, many health workers and other health system staff noted their commitment and intrinsic motivation to their roles [26].

### 3.3. Local Solutions Address Power Imbalances

Local co-creation workshops succeeded in reconceptualizing the problem of no immunization and under-immunization among participants, by presenting challenges from caregivers’ or healthcare workers’ perspectives. The exercise challenged each participant to empathize with caregivers and healthcare workers by better understanding the barriers they face in getting children vaccinated. By facilitating group discussions with caregivers and health workers, it allowed all community and health system participants in the workshop to work together to identify how they were responsible in supporting caregivers to overcome barriers. It was a new experience for all participants to be brought into a workshop where district, health facility, community leaders, and caregivers were invited as equals. In the DRC and Mozambique, district and provincial stakeholders expressed that the workshops were enlightening, and their perspectives changed about mothers related to the barriers they face and their agency in overcoming them.

Community-level participants from all countries expressed excitement at how they could support caregivers in getting their children to the health center. Solutions that emerged from the workshop included forming walking groups of caregivers to travel together to health facilities (Mozambique), husbands helping with transport (Mozambique) or childcare (Nigeria), and championship by community and religious leaders, who themselves are supported with training and information (DRC and Mozambique). These solutions suggest that participants were motivated by feelings of social cohesion. All co-creation workshops also proposed better implementation of existing solutions, such as outreach vaccination. Health system participants often uncovered new knowledge about financing challenges between levels of the health system that inhibited their ability to better support healthcare workers in adhering to their facility’s immunization goals or implement outreach activities. Multiple solutions reflected ideas of people-centered care and improved service experience, such as joint planning for outreach services across health programs to better reach remote communities, integrated delivery of all child health services at facilities, reducing waiting times, and expanded service hours.

## 4. Discussion

The drivers of vaccination, identified through this study, are consistent with other studies, but we provide a new way of reconceptualizing them through HCD and intersectional lenses. Viewed through a caregivers’ perspective, each individual has a unique set of social, institutional, and structural circumstances that intersect and interact to constrain or enable her options and outcomes. As Crenshaw argued when she proposed the intersectionality theory [27], it is limiting to group caregivers into binary categories, such as race or gender or to attribute a single characteristic—such as religion, education, or wealth—to explain immunization inequalities. A complete understanding of the drivers of inequality requires analyzing the joint influence of multiple factors related to the individual, as well as the health system and greater structural context. Our study used qualitative interviews guided by an HCD mindset to identify the lived experiences, challenges, and needs of caregivers of un- and under-vaccinated children. Presentation of their stories in community-level workshops built empathy and enabled co-design of locally relevant solutions that addressed the needs and preferences of caregivers. Some of the solutions were novel to the researchers, such as community walking groups, but many were in fact the improved implementation of existing strategies, such as outreach vaccination. As a project with the goal of overcoming entrenched obstacles to immunization equity, the resulting solutions guided our choice of activities and their design, with a focus on strengthening the local capacity for gender integration, strengthening community partnerships, and addressing root causes of sub-optimal service experience.

Our empirical data demonstrated the lack of person-centeredness or alignment of immunization programs with client needs, particularly for caregivers and communities facing multiple intersecting vulnerabilities.

Despite the many pro-equity strategies that exist and are budgeted and planned for [28], very few were actually implemented due to financial resource constraints at the operational level stemming from weak accountability and governance. We note that strategies to reach zero-dose children likely cost more, and that at the operational level, vaccinating individuals and communities at a higher risk of morbidity and mortality should be prioritized [29]. With the increased global investment in pro-equity strategies to reach zero-dose children and missed communities, we note the importance of also strengthening accountability for implementation and stronger health system governance and management.

Another root cause of the lack of people-centeredness stems from the way in which power structures are entrenched in health systems. On an interpersonal level, this can result in inconvenient or disrespectful services, but on an institutional level, results in weak accountability and insufficient resources to improve access, quality and experience. We saw evidence that stakeholders at operational and community levels were interested in and committed to taking actions to support caregivers to access vaccination, ensure the implementation of outreach vaccination services, and improve the overall convenience of services. Will they succeed? We believe this is the level where efforts to reorient PHC around user needs can have the most traction, although tangible pathways towards improved empathy and person-centeredness exist also at planning, policy and funding levels. Policies and programs can invest in or encourage approaches that are gender responsive, people-centered, integrate HCD and intersectional lenses, and explicitly address institutional and structural root causes. For example, tools for operational planning, such as integrated microplans, can be revised to ensure identification of the barriers and needs of the hardest-to-reach, and can engage caregivers and communities in the identification of solutions. Technical partners, such as our project, can catalyze the engagement of non-traditional partners to fill resource gaps needed to implement pro-equity strategies (e.g., local businesses) and ensure accurate sharing of information from trusted voices (e.g., religious leaders). Policymakers and external funders can support efforts to integrate the delivery of all PHC services for improved efficiency and client satisfaction, address human resource motivation, and improve management skills.

### 4.1. Comparison with Other Research

This study identified similar determinants of vaccine inequality as has been observed across other qualitative and quantitative research on drivers of immunization coverage and equity, including access, cost, health systems readiness, gender-related barriers, vaccine supply, fear of side effects, community engagement, lack of knowledge, and provider absenteeism [3,4,5,10]. Our study contributes to this literature by identifying the relationships among these barriers and how those interactions contribute to no vaccination or under-vaccination. As noted in the recent systematic review of vaccination barriers by Kaufman [7], less than half of all global studies report barriers across all determinants of vaccination, but our study was designed to holistically and comprehensively explore barriers from the perspective of caregivers. A handful of published studies use HCD approaches to study vaccination barriers and solutions, including ones from Mozambique [19], India [30], and the Philippines [31]. The Mozambique study identified similar patterns of barriers to vaccination, including the role of gender barriers and power imbalances with health workers. Cross-national quantitative analyses of household survey data show that immunization inequalities are associated with household wealth and maternal education [6], and that the prevalence of zero-dose children is associated with gender inequality [8], birth order, birth weight, maternal education, maternal occupation, household wealth, and the number of antenatal care visits [9].

### 4.2. Limitations

Our study had some limitations related to the design and implementation. The intentional selection of study units with high proportions of zero-dose children means that our findings are not necessarily representative of the countries, or even states/provinces within the countries, and these findings should not be interpreted to represent the most common or typical barriers to vaccination, but rather the barriers faced by those most excluded from access to quality immunization services. Because we sought to tailor the study design for the context of each country, comparing or synthesizing the findings across countries should be treated with caution. We did not originally design the study with the intersectionality lens in mind, and as such, we missed the opportunity to explore specific intersections and interactions during data collection. We were able to identify and interview caregivers of zero-dose children in most study settings, but not the Edo province in Nigeria. Similarly, the study was not implemented in regions with refugees, displacement, or conflict-affected populations, which we know face many barriers to vaccination. We designed the study to be implemented rapidly to inform timely program design, but the short duration of the data collection period limited the number of respondents interviewed in each community, which may mean the findings are biased. In Nigeria, we used FGDs instead of in-depth interviews with caregivers and community members to optimize the limited time available, but FGDs may have consequences on the type of information shared, particularly for sensitive information. Similarly, because we prioritized the ability to validate most findings in co-creation workshops immediately following data collection, we did not have time to analyze quantitative data during the rapid study period.

## 5. Conclusions

This qualitative study presents drivers of immunization inequality from the perspectives of caregivers, who face multiple, often compounding, barriers related to social, institutional, and structural dynamics. Applying HCD and intersectional approaches highlights the little agency caregivers face in their journeys to vaccinate their children, and how vaccination and PHC services are not designed with their needs in mind. We found that caregivers who face the greatest number of barriers to accessing and receiving quality immunization services tend to face a double burden of living in communities where outreach vaccination strategies are unimplemented due to weak governance and accountability. Based on our experiences, implementing this study and our observation that it was feasible to build empathy and co-design solutions with caregivers, communities, and health system actors, we recommend the integration of HCD and intersectionality approaches and tools in immunization research and programs. Immunization and PHC professionals at all levels can take simple steps to integrate HCD and intersectionality into their planning, management, and implementation processes, such as:

- As part of routine coverage and equity analyses that many countries undertake [32], select qualitative methods that engage directly with caregivers and communities and work with them to identify locally relevant solutions.
- Revise existing planning processes (e.g., annual planning processes, funding applications) and tools (e.g., microplanning tools, supervision checklists) to provide guidance or requirements related to gender integration, engaging communities, and addressing root causes of sub-optimal implementation.
- Invest in strengthening skills and culture related to gender, intersectionality, and HCD among immunization stakeholders to ensure strategies, activities, and interventions address the needs of the most vulnerable caregivers and families.
- Encourage donors, such as Gavi, to target investments towards interventions that are gender-responsive or transformative, towards activities that are designed to reach caregivers and communities furthest from health justice, and towards supporting larger health systems and governance reforms that improve the availability, quality, and convenience of people-centered PHC approaches.
- Encourage and fund research and evaluation of the effectiveness and equity consequences of existing and new interventions to reach zero-dose children and missed communities [28], and on how to overcome entrenched obstacles related to their implementation.

We found that the power of local knowledge must be leveraged as a catalyst for all of these steps.

## Figures and Tables

**Table 1:** HCD tools used during co-creation workshops.

HCD Tool	Brief Description	Purpose
Mothers’ Vaccination Ecosystem	Visual bullet eye diagram to represent a mother (or female caregiver) and her baby, and the barriers she faces at each ecosystem level: family, community, health system. Used as a discussion and group-work tool.	To generate empathy for mothers by illustrating the many intersecting barriers they face. To encourage responsibility and collective action by other stakeholders by seeing and discussing barriers faced by mothers, and generate ideas to reduce barriers mothers face.
Solution Brief	The solution brief is a persona tool that draws data from synthesized findings from in-depth interviews and focus group discussions. Drawing from the data, the tool includes a persona (a fictionalized representation of an individual; see Table 2), assumptions about the root causes of immunization barriers, and problem or opportunity statements for specific user personas, which could include caregivers, health workers, or others. The end of the brief outlines a specific problem, or solution framing in the form of a question to address; this is the beginning point of the solutioning activity.	To generate empathy for specific users (e.g., healthcare workers, caregivers, community health workers). To simplify the complex system of barriers to immunization by seeing the barriers from the perspective of a specific user.
Solutioning Activity	This solutioning activity tool is a collaborative problem-solving exercise for small group breakout teams. Each team receives a solution brief detailing the user persona and their challenges, as well as a specific problem for the group to address, based on the user’s perspective. As a group often including users who share this challenge, the team works through a set of prompts from brainstorming, to using a rubric to decide which ideas to build out, to finally building out two solutions for the user’s problem.	To generate multiple-solution ideas to a specific immunization barrier in a short period of time. To build the relationship between community and health system by working toward shared solutions. Triangulate data collected from in-depth interviews and focus groups.

**Table 2:** Select caregiver personas used during co-creation workshops, based on study findings.

Study Theme	Problem Statements from Solution Briefs/Personas
Theme 1: Caregiver facing multiple intersecting and interacting barriers based on her context	When mother was able to get to the health center, she was not provided vaccines because she did not have a vaccination card. Mother did not have money for a card (although these should be free, she was told she would need to pay for one). Mother was insulted by a nurse for having a baby outside of the health center, but she had no way to get to the health center for her birth, as it was 35 km away.
Theme 2: Immunization services not aligned with a caregivers’ needs	Mother has missed vaccinations due to lack of time (she was busy with household chores). She makes decisions about her child’s health on her own, but has the support/advice of her husband, and he is the one who takes them to the health facility. Mother received advice on child health from her aunt. Mother heard about the mobile brigade through the religious leader. Mother regrets the fact that the mobile vaccination services sometimes postpone, and when this happens mothers are not informed in advance; she had already gone to the scheduled place and date in vain. Unlike the other mothers, she preferred the mobile vaccination services to be in the morning, so she can dedicate the rest of the day to her errands.

**Table 3:** Study participants.

		Number of Participants by Type and Country		
Health System Level	Participant Type	DRC (Kasaï (Ndjoko Punda, Kalonda Ouest); Kasaï Oriental (Nzaba, Cilundu); Lualaba (Dilala, Kazenze))	Mozambique (Zambezia (Gurue, Ile, Molumbo) Nampula (Murrupula, Erati, Mossuril))	Nigeria (Edo (Ikpoba Okha) and Jigawa (Buji))
Community	Caregivers	24	20	18 (FGDs)
	Community leaders and activists; community health workers and volunteers; traditional birth attendants	12	25	10 (FGDs)
Health facility	Facility-level healthcare worker; vaccinators; nurse managers	12	10	8
District/health zone	District/zonal EPI manager, community engagement focal point, monitoring and evaluation focal point, financial officer, health Education officer	6	11	14
Province	Provincial logistics focal point, EPI manager, MEL, finance officer, community engagement/health education focal point	6	6	16
	TOTAL	60	72	66

FGD: focus group discussion; EPI: Expanded Program on Immunization; MEL: Monitoring, Evaluation, and Learning.

**Table 4:** Examples of findings through the intersectionality lens.

Intersection	Resulting Interaction	Impact on Vaccination Outcomes, Particularly Zero-Dose Children
Gender × wealth	Caregivers with fewer resources were less able to overcome gender barriers related to their paid or unpaid work and opportunity costs of going to the health facility.	These caregivers were more likely to have zero-dose children than other caregivers.
Gender × geographic access	Caregivers felt unsafe traveling to distant health facilities, up to 2 days in Mozambique, and did not have resources for other forms of transport.	Many caregivers of zero-dose children cited this barrier, and these also comprise missed communities.
Wealth × service experience	Caregivers of low socioeconomic status faced more disrespectful care.	Typically resulted in under-vaccination, where caregivers who were treated poorly did not return for additional vaccines.
Wealth × governance × financing	Caregivers who could not afford fees for services or cards could not access vaccination services. These fees were often charged as a substitute for formal income.	Many caregivers of zero-dose children cited this barrier.
Wealth × geographic access × financing	Caregivers in the most remote and poorest communities were least likely to be reached by outreach vaccination services.	Many caregivers of zero-dose children cited this barrier.

## Data Availability

The data presented in this study are available on request from the corresponding author. The data are not publicly available due to privacy concerns of the respondents.
